# Supplementary material for: Change in PD-L1 and CD8 Expression after Chemoradiotherapy for Esophageal Squamous Cell Carcinoma
Source: Biomedicines. 2022 Aug 4;10(8):1888. doi: 10.3390/biomedicines10081888 (PMC9405329; doi:10.3390/biomedicines10081888)
Supplement: Supplementary file 1 [file biomedicines-10-01888-s001.zip › biomedicines-1836775-supplementary.pdf]

Supplementary Material

This appendix is provided by the authors to give readers additional information about this study.

Change in PD-L1 and CD8 expression after chemoradiotherapy for esophageal squamous cell carcinoma

Wei-Chung Chen, Chun-Chieh Wu, Yi-Hsun Chen, Jui-Ying Lee, Yao-Kuang Wang, Nian-Siou Wu, Ming-Tsang Wu, I-Chen Wu

Table S1 - Table S5

Figure S1 - Figure S2

References

Table S1. Clinicopathological characteristics of subgroup ESCC patients

[a]These patients did not provide the tumor grade in pathological reports.

Characteristics	With paired specimens N=23 Mean ±SD or No. (%)		Without paired specimens N= 41 Mean ±SD or No. (%)	
**Gender**				
Male	22	(95.7)	39	(95.1)
Female	1	(4.3)	2	(4.9)
**Age (years)**	55.97	±8.62	56.14	±8.01
**Pathologic status**				
Stage I	0	(0)	10	(24.4)
Stage II	0	(0)	14	(34.1)
Stage III	22	(95.7)	14	(34.1)
Stage IV	1	(4.3)	3	(7.3)
**Tumor differentiation**				
Grade 1 (Well)	3	(13.0)	2	(4.9)
Grade 2 (Moderate)	15	(65.2)	34	(82.9)
Grade 3 (Poor)	3	(13.0)	3	(7.3)
Missing[a]	2	(8.7)	2	(4.9)
**Treatment**				
ESD	0	(0)	5	(12.2)
OP	0	(0)	14	(34.1)
CCRT	7	(30.4)	18	(43.9)
CCRT then OP	16	(69.6)	4	(9.8)
**CCRT Response**				
Without CCRT	0	(0)	19	(46.3)
Complete response	0	(0)	7	(17.1)
Partial response	22	(95.7)	10	(24.4)
Stable disease	1	(4.3)	4	(9.8)
Progressive disease	0	(0)	1	(2.4)
**PD-L1 H-score**				
H-score Median < 2	11	(47.8)	18	(43.9)
H-score Median ≥ 2	12	(52.2)	23	(56.1)

Abbreviations: ESD, Endoscopic submucosal dissection; CCRT, Concurrent chemoradiation therapy; CT, chemotherapy; OP, esophageal resection surgery

Table S2. The H-score distribution of 64 patients.

H-score	Numbers (n=64)	Percentage
0	16	25.00%
1	13	20.31%
2 (Median)	6	9.38%
4	1	1.56%
5	2	3.13%
8	1	1.56%
10	2	3.13%
12	1	1.56%
15	2	3.13%
19	1	1.56%
20	1	1.56%
25	2	3.13%
30	2	3.13%
45	1	1.56%
70	1	1.56%
75	2	3.13%
80	1	1.56%
90	1	1.56%
100	1	1.56%
105	1	1.56%
110	1	1.56%
150	1	1.56%
160	1	1.56%
170	1	1.56%
195	1	1.56%
200	1	1.56%

Table S3. Multivariate analysis of factors associated with overall survival in the 23 patients with paired specimens across CCRT by PD-L1 up and down regulation.

Variables		No.	Adjusted HR	95% CI	P value
**Post CCRT Minus Pre CCRT**	PD-L1 H score				
	Down-regulated	10	1		
	Stable or Up-regulated	13	0.466	0.163-1.133	0.154
	Gender				
	Male	22	1		
	Female	1	0.000	0.00	0.990
	Age	23	1.049	0.983-1.119	0.147

Abbreviations: HR, Hazard Ratio; CI, confidence interval

Table S4. Multiple logistic regression analysis of factors associated with CD8 density in 15 patients before and after CCRT.

Variables	No.	CD8 density (High vs Low)[a] adjusted HR	95% CI	P value
**PD-L1 H score (Pre CCRT)**				
< 2	10	1		
≥ 2	5	0.36	0.03-3.95	0.40
**Gender**				
Male	14	1		
Female	1	--		
**Age**	15	0.97	0.83-1.13	0.72
**PD-L1 H score (Post CCRT)**				
< 2	10	1		
≥ 2	5	1.32	0.13-13.92	0.82
**Gender**				
Male	14	1		
Female	1	--		
**Age**	15	0.98	0.85-1.13	0.81

[a]Median value of CD8 density was used as cutoff. Pre CCRT group: <94.2 vs ≥94.2; Post CCRT group: <149.2 vs ≥149.2

Abbreviations: HR, Hazard Ratio; CI, confidence interval; CCRT, concurrent chemoradiation therapy

Table S5. Literature review of PD-L1 expression rate in ESCC studies.

Patient numbers	Ethnicity	PD-L1 expression rate	PDL1_Overall Survival (OS)	Publications
536	Chinese	117/349 (33.5%)	Survival analysis (poor prognosis, P = 0.047)	(1)
162	Chinese	74/162 (45%)	Survival analysis (poor prognosis, P = 0.000);<br>Multivariate analysis (poor prognosis, OR=0.380, 95%CI=0.200-0.648, P = 0.001)	(2)
233	Chinese	129/233 (55.4%)	Survival analysis (good prognosis, P = 0.023);<br>Multivariate analysis (good prognosis, HR=0.697, 95%CI=0.498-0.976, P = 0.035)	(3)
246	Chinese	60/246 (24.4%)	Survival analysis in OP only (not significant, P = 0.706);<br>Survival analysis in post chemotherapy (poor prognosis, P = 0.765);<br>Survival analysis in post radiotherapy (poor prognosis, P = 0.047);<br>Survival analysis in post CCRT (poor prognosis, P = 0.061)	(4)
106	Chinese	57/106 (46.2%)	Survival analysis (poor prognosis, P = 0.027)	(5)
338	Chinese	113/378 (29.9%)	Survival analysis (not significant, P = 0.140);<br>Multivariate analysis (not significant, HR= 0.882, 95%CI = 0.648–1.200, P = 0.423)	(6)
146	Chinese	90/146(61.7%)	Survival analysis (poor prognosis, P = 0.010);<br>Multivariate analysis (poor prognosis, HR= 1.643, 95%CI = 1.038–	(7)

			2.601, P = 0.034)	
133	Chinese	56/133 (42.1%)	Survival analysis (poor prognosis, P = 0.01);<br>Multivariate analysis (poor prognosis, HR=1.957, 95%CI: 1.303–2.939, P = 0.001)	(8)
279	Chinese	Intraepithelial: 74.28%; Tumor: 32.59%	Survival analysis (poor prognosis, P = 0.046)	(9)
138 (82 had paired pre- and post-CRT)	Chinese	57/138 (41.3%)	Survival analysis (not significant, P = 0.133);<br>Univariate analysis (not significant, HR=0.642, 95%CI: 0.360–1.145, P = 0.133)	(10)
41	Japanese	qPCR: 15/31 (48.4%); IHC: 13/31 (41.9%)	Survival analysis (poor prognosis, P = 0.025);<br>Multivariate analysis (poor prognosis, P = 0.0001 without detailing data)	(11)
90	Japanese	17/91(18.9%)	Survival analysis (poor prognosis, P = 0.027);<br>Multivariate analysis with HLA class I (poor prognosis, HR=2.95, 95% C=1.03-7.50, P = 0.0447)	(12)
111 with chemotherapy 69 without chemotherapy	Japanese	53/180 (29.4%)	Survival analysis (poor prognosis, P =0.0010);<br>Survival analysis with chemotherapy (poor prognosis, P = 0.0064);<br>Multivariate analysis (poor prognosis, HR= 1.7480, 95%CI = 1.1373–2.6578, P = 0.0114);	(13)
90	Japanese	57/90 (63.3%)	Survival analysis (poor prognosis, P = 0.0112);	(14)

			Multivariate analysis (poor prognosis, HR=1.957, 95%CI: 1.303–2.939, P = 0.001)	
76	Japanese	Tumor 30/76(39.5%); Stroma 39/76(51.3%)	Survival analysis in tumor (not significant, P = 0.84);<br>Survival analysis in stroma (good prognosis, P = 0.04);<br>Multivariate analysis in stroma (good prognosis, HR= 0.45, 95%CI = 0.23-0.85, P <0.05)	(15)
19 with pre- and post-CRT paired tissue<br>9 with Chemotherapy alone<br>45 with only Post-CRT tissue	Korean	41/73(56.2%)	Survival analysis (poor prognosis, P = 0.020);<br>Multivariate analysis (poor prognosis, HR= 2.29, 95%CI = 1.12–4.69, P = 0.023)	(16)
200	Korean	67/200(33.5%)	Survival analysis (not significant, P =0.656)	(17)
58	Korean	CPS>=1 29/51 (56.9%); CPS>=10, 14/51 (27.5%); CPS>=20, 9/51 (17.6%)	Better OS trend in CPS >=10 (Not significant)	(18)

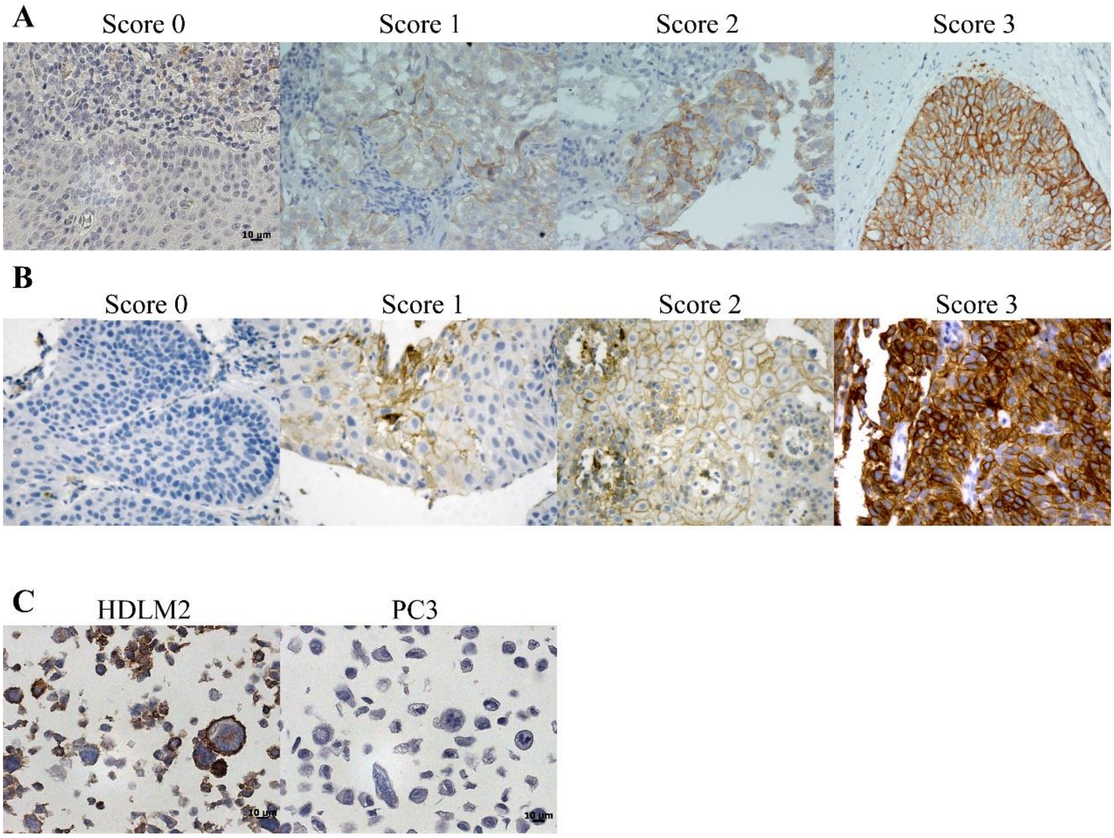

Figure S1. Scoring criteria of PD-L1 immunohistochemistry staining and antibody specificity validation. All images were taken under 200 × magnification. A) Representative images of membrane intensity score by E1L3N clone.; B) Representative images of membrane intensity score by 22C3 clone.; C) The presence and absence of anti-PD-L1 antibody E1L3N clone signals in positive control HDLM2 cells, and in negative control PC3 cells

A

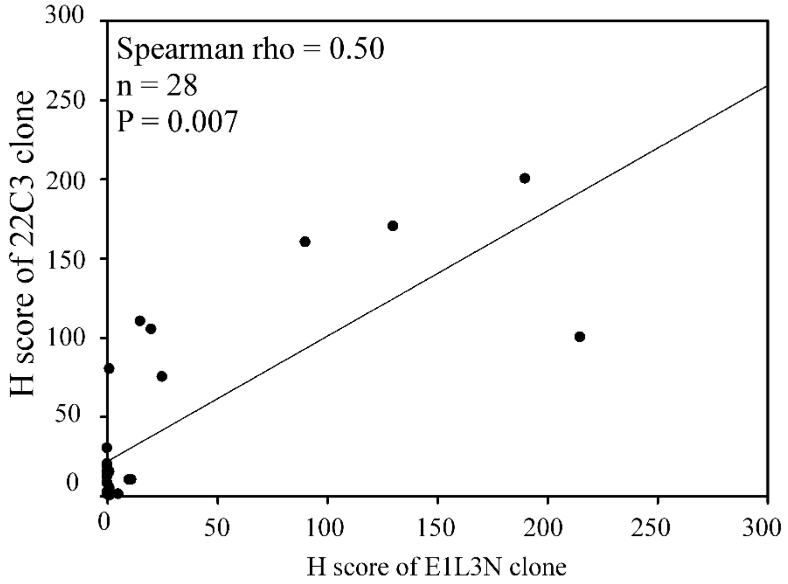

B

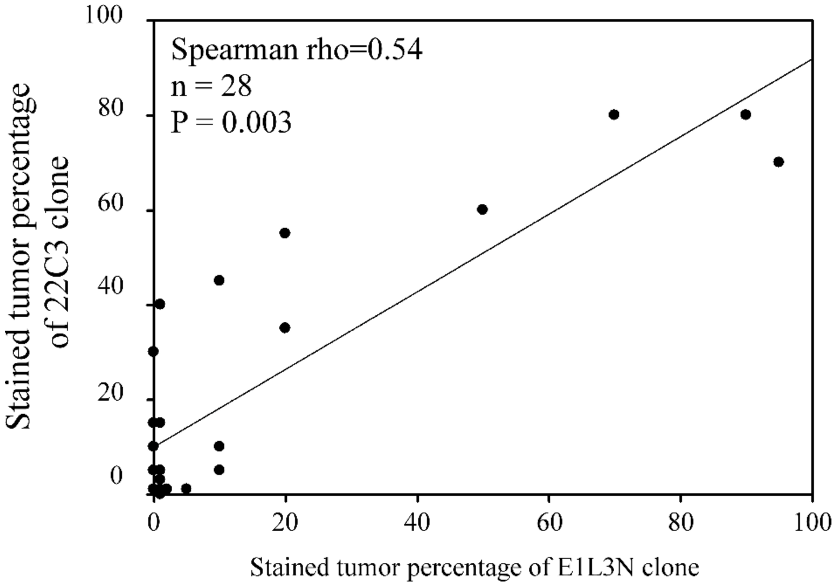

Figure S2. Analysis of PD-L1 antibody sensitivity from 28 specimens. A) Scatter plot comparing the H scores between E1L3N and 22C3 clone;

B) Scatter plot comparing the stained tumor proportion between E1L3N and 22C3 clone

References

1. Chen K, Cheng G, Zhang F, Zhang N, Li D, Jin J*, et al.* Prognostic significance of programmed death-1 and programmed death-ligand 1 expression in patients with esophageal squamous cell carcinoma. Oncotarget **2016**;7(21):30772-80 doi 10.18632/oncotarget.8956.
2. Chen MF, Chen PT, Chen WC, Lu MS, Lin PY, Lee KD. The role of PD-L1 in the radiation response and prognosis for esophageal squamous cell carcinoma related to IL-6 and T-cell immunosuppression. Oncotarget **2016**;7(7):7913-24 doi 10.18632/oncotarget.6861.
3. Guo W, Zhang F, Shao F, Wang P, Li Z, Yang X*, et al.* PD-L1 expression on tumor cells associated with favorable prognosis in surgically resected esophageal squamous cell carcinoma. Hum Pathol **2019**;84:291-8 doi 10.1016/j.humpath.2018.09.014.
4. Jiang C, Zhu Y, Tang S, Zhang G, Lin Q, Xu Y*, et al.* High PD-L1 expression is associated with a favorable prognosis in patients with esophageal squamous cell carcinoma undergoing postoperative adjuvant radiotherapy. Oncol Lett **2019**;17(2):1626-34 doi 10.3892/ol.2018.9747.
5. Leng C, Li Y, Qin J, Ma J, Liu X, Cui Y*, et al.* Relationship between expression of PD-L1 and PD-L2 on esophageal squamous cell carcinoma and the antitumor effects of CD8(+) T cells. Oncol Rep **2016**;35(2):699-708 doi 10.3892/or.2015.4435.
6. Rong L, Liu Y, Hui Z, Zhao Z, Zhang Y, Wang B*, et al.* PD-L1 expression and its clinicopathological correlation in advanced esophageal squamous cell carcinoma in a Chinese population. Diagn Pathol **2019**;14(1):6 doi 10.1186/s13000-019-0778-4.
7. Wang Q, Feng F, Wang F, Liu Z, Liu S, Xu G*, et al.* PD-L1 Expression On tumor Cells Was Associated With Unfavorable Prognosis In Esophageal Squamous Cell Carcinoma. J Cancer **2018**;9(12):2224-31 doi 10.7150/jca.24493.
8. Zhu Y, Li M, Mu D, Kong L, Zhang J, Zhao F*, et al.* CD8+/FOXP3+ ratio and PD-L1 expression associated with survival in pT3N0M0 stage esophageal squamous cell cancer. Oncotarget **2016**;7(44):71455-65 doi 10.18632/oncotarget.12213.
9. Pan C, Wang Y, Liu Q, Hu Y, Fu J, Xie X*, et al.* Phenotypic profiling and prognostic significance of immune infiltrates in esophageal squamous cell carcinoma. Oncoimmunology **2021**;10(1):1883890 doi 10.1080/2162402X.2021.1883890.
10. Zhou S, Yang H, Zhang J, Wang J, Liang Z, Liu S*, et al.* Changes in Indoleamine

2,3-Dioxygenase 1 Expression and CD8+ Tumor-Infiltrating Lymphocytes after Neoadjuvant Chemoradiation Therapy and Prognostic Significance in Esophageal Squamous Cell Carcinoma. Int J Radiat Oncol Biol Phys **2020**;108(1):286-94 doi 10.1016/j.ijrobp.2020.01.020.

11. Ohigashi Y, Sho M, Yamada Y, Tsurui Y, Hamada K, Ikeda N*, et al.* Clinical significance of programmed death-1 ligand-1 and programmed death-1 ligand-2 expression in human esophageal cancer. Clin Cancer Res **2005**;11(8):2947-53 doi 10.1158/1078-0432.CCR-04-1469.

12. Ito S, Okano S, Morita M, Saeki H, Tsutsumi S, Tsukihara H*, et al.* Expression of PD-L1 and HLA Class I in Esophageal Squamous Cell Carcinoma: Prognostic Factors for Patient Outcome. Ann Surg Oncol **2016**;23(Suppl 4):508-15 doi 10.1245/s10434-016-5376-z.

13. Tanaka K, Miyata H, Sugimura K, Kanemura T, Hamada-Uematsu M, Mizote Y*, et al.* Negative influence of programmed death-1-ligands on the survival of esophageal cancer patients treated with chemotherapy. Cancer Sci **2016**;107(6):726-33 doi 10.1111/cas.12938.

14. Tsutsumi S, Saeki H, Nakashima Y, Ito S, Oki E, Morita M*, et al.* Programmed death-ligand 1 expression at tumor invasive front is associated with epithelial-mesenchymal transition and poor prognosis in esophageal squamous cell carcinoma. Cancer Sci **2017**;108(6):1119-27 doi 10.1111/cas.13237.

15. Ito N, Tsujimoto H, Horiguchi H, Shimazaki H, Miyazaki H, Saitoh D*, et al.* Clinical Significance of Programmed Death Ligand-1 Expression in Esophageal Squamous Cell Carcinoma. J Surg Res **2020**;251:321-8 doi 10.1016/j.jss.2020.02.013.

16. Lim SH, Hong M, Ahn S, Choi YL, Kim KM, Oh D*, et al.* Changes in tumour expression of programmed death-ligand 1 after neoadjuvant concurrent chemoradiotherapy in patients with squamous oesophageal cancer. Eur J Cancer **2016**;52:1-9 doi 10.1016/j.ejca.2015.09.019.

17. Kim R, Keam B, Kwon D, Ock CY, Kim M, Kim TM*, et al.* Programmed death ligand-1 expression and its prognostic role in esophageal squamous cell carcinoma. World J Gastroenterol **2016**;22(37):8389-97 doi 10.3748/wjg.v22.i37.8389.

18. Lee J, Kim B, Jung HA, La Choi Y, Sun JM. Nivolumab for esophageal squamous cell carcinoma and the predictive role of PD-L1 or CD8 expression in its therapeutic effect. Cancer Immunol Immunother **2021**;70(5):1203-11 doi 10.1007/s00262-020-02766-7.
